# Gender and Social Inequalities in Awareness of Coronary Artery Disease in European Countries

**DOI:** 10.3390/ijerph19031388

**Published:** 2022-01-26

**Authors:** Antonio Daponte-Codina, Emily C. Knox, Inmaculada Mateo-Rodriguez, Amanda Seims, Vera Regitz-Zagrosek, Angela H. E. M. Maas, Alan White, Floris Barnhoorn, Fernando Rosell-Ortiz

**Affiliations:** 1 Andalusian School of Public Health, 18011 Granada, Spain; antonio.daponte.easp@juntadeandalucia.es; 2 CIBER Epidemiology and Public Health (CIBERESP), 28029 Madrid, Spain; emily.knox.easp@juntadeandalucia.es; 3 Department of Social and Organizational Psychology, Faculty of Psychology, Universidad Nacional de Educación a Distancia (UNED), 28040 Madrid, Spain; 4 Bradford Teaching Hospitals NHS Foundation Trust, Bradford BD9 6RJ, UK; amanda.seims@bthft.nhs.uk; 5 Department of Cardiology, University of Zurich, CH-8006 Zurich, Switzerland; vera.regitz-zagrosek@charite.de; 6 Charité, Universitätsmedizin Berlin, 10117 Berlin, Germany; 7 Institute for Gender Medicine, German Centre for Cardiovascular Research, Partner Site Berlin, 10785 Berlin, Germany; 8 Department of Cardiology, Radboud University Medical Center, 6500 HB Nijmegen, The Netherlands; Angela.Maas@radboudumc.nl; 9 School of Health and Community Studies, Leeds Beckett University, Leeds LS6 3QS, UK; alan@alanwhitemenshealth.com; 10 European Public Health Association (EUPHA), 3500 BN Utrecht, The Netherlands; f.barnhoorn@euphaoffice.org; 11 Medical Emergency Services 061, 26580 La Rioja, Spain; fernandorosell@gmail.com

**Keywords:** coronary artery disease, awareness, gender bias, sex differences, inequalities, Europe

## Abstract

Coronary artery disease (CAD) is the single leading cause of death in Europe and the most common form of cardiovascular disease. Little is known about awareness in the European population. A cross-sectional telephone survey of 2609 individuals from six European countries was conducted to gather information on perceptions of CAD, risk factors, preventive measures, knowledge of heart attack symptoms and ability to seek emergency medical care. Level of awareness was compared according to gender, age, socioeconomic status (SES) and educational level. Women were approximately five times less likely than men to consider heart disease as a main health issue or leading cause of death (OR = 0.224, 95% CI: 0.178–0.280, OR = 0.196, 95% CI: 0.171–0.226). Additionally, women were significantly less likely to have ever had a cardiovascular screening test (OR = 0.515, 95% CI: 0.459–0.578). Only 16.3% of men and 15.3% of women were able to spontaneously identify the main symptoms of a heart attack. Almost half of the sample failed to state that they would call emergency services in case of a cardiac event. Significant differences according to age, SES and education were found for many indicators amongst both men and women. Development of a European strategy targeting improved awareness of CAD and reduced gender and social inequalities within the European population is warranted.

## 1. Introduction

Coronary artery disease (CAD) accounts for 20% of all mortality in Europe and is the most common form of cardiovascular disease (CVD). According to the latest data, CAD represents 16% of all premature mortality under 75 years old among women and 18% among men [1]. Furthermore, there are large differences between countries, with some European countries having almost 10 times greater rates that others in both men and women [2]. These large inequalities indicate that there is great room for improvement when it comes to reducing the burden of CAD in European countries.

CAD is largely due to a limited number of risk factors, including certain behaviors [3]. It is widely recognized that improving behaviors such as smoking, following a healthy diet or engaging in physical activity can significantly reduce CHD incidence and mortality [4]. Awareness of cardiovascular disease is a hugely important factor when it comes to individuals making lifestyle changes, adopting preventive measures, and complying with health recommendations and treatment guidelines [5]. Furthermore, awareness of personal risk has been associated with the adoption of secondary prevention measures by patients, although there is a need to improve understanding of the association between health perceptions and cardiovascular preventive behaviors [6,7]. Increasing awareness is effective for improving the timely and appropriate use of different levels of health services following coronary events [8]. In short, a degree of basic knowledge about risk factors, and prevention and protection against CAD within the general population is essential for improving cardiovascular health [9,10]. The recent COVID-19 crisis has emphasized once more that education, socio-economic status (SES), living conditions and ethnicity are crucial factors in health and this also accounts for CAD [11]. Moreover, chronic stress induced by air pollution and traffic noise are now also well recognized as important determinants of cardiovascular health [12]. 

It is scientifically well established that there are important differences between men and women at all stages of the CAD process, from risk factors at the prehospital phase to diagnosis, treatment, rehabilitation and outcomes [5,13]. Moreover, some studies have shown that women lack awareness of CAD risk factors and symptoms, the impact of CAD on their health, measures to be taken during a coronary crisis, how to request medical assistance and options regarding transportation to hospital [14,15].

Furthermore, very few studies have addressed population awareness of CAD in Europe, in contrast with the long tradition of such research seen in other countries [10]. For all these reasons, the GenCAD project (gender specific mechanisms in coronary artery disease) was developed. This project was aimed at improving understanding of sex and gender differences in coronary artery disease in European countries. 

Within the framework of this project, we carried out a study aimed at determining levels of awareness within the European population regarding essential aspects of CHD. 

## 2. Materials and Methods

A cross-sectional survey was conducted of 2609 individuals from six European countries from November 2017 to March 2018. 

We conducted an extensive review of the scientific literature, searching for studies on awareness of CAD and cardiovascular disease within the general population. Based on the questionnaires of selected studies [5,9,15], we developed a questionnaire which included questions about coronary heart disease, specifically, perceptions of its relevance for health, risk factor identification and lifestyle behaviors, preventive measures, knowledge of heart attack symptoms (defined as myocardial infarction or angina pectoris) and capacity to seek out appropriate emergency medical care. Data were also collected on basic demographic and socioeconomic details such as sex, age, income and educational level.

The questionnaire included mostly closed questions, with the exception of questions on the following topics: CAD as a leading health issue and leading cause of death, heart attack symptoms and first reaction to suffering a hypothetical heart attack, and main risk factors related to CVD. Questions on these areas were open-ended, allowing the spontaneous responses of participants to be collected.

The questionnaire was validated through focus groups according to a series of selected criteria in order to evaluate the appropriateness and quality of questions. The final version of the questionnaire was translated into the six languages corresponding to the participating countries. Experts in cardiology and other medical specialties, psychology, sociology, survey development and public health, from the institutions collaborating on the project carried out this entire process. The complete questionnaire is attached online as a supplementary file.

Respondents were aged at least 25 years old and came from Bulgaria, Croatia, Czech Republic, Germany, Spain and Sweden. These countries were selected to represent the great diversity among European Member States with regards to key indicators such as CHD epidemiology, economic level, population size, and geographic location.

An estimated sample size of 2600 individuals was calculated to give an overall margin of error of +/−1.96% according to the assumption of maximum non-determination and with a confidence level of 95%. Given the large differences in population size between the selected countries, sample distribution between countries was not proportional to their respective population sizes.

Within each country, sample distribution was representative of regional variations in population size and type (towns, cities, etc.). Selection of the final sample was performed according to gender and age quotas, specific to each locality and region. A global market research company, formerly named TNS (currently Kantar), was subcontracted to adapt the questionnaire into a telephone survey, translate it into the language of each target country and conduct interviews. Within each country, households were selected at random using random digit dialing. Calls were made to both landline and mobile phones in order to maximize representativeness. Consent to participate was verbally obtained from participant subjects, after informing them of the study objectives and procedures. If a participant refused to answer or did not know how to answer a particular question, responses were coded as “don’t know” or “no answer”. These participants were not excluded from the analysis.

Data were also weighted to adjust for differences in the population sizes of the six countries. In this way, data reflected the European population of individuals aged 25 years and older. Further, it was noted that intra-individual income differences were not comparable between countries, e.g., A monthly income of 1000 € is extremely high in Bulgaria but extremely low in Sweden. Thus, the socioeconomic variable was transformed to adjust for inter-country differences relating to income, with participants’ SES being classified as ‘high’ or ‘low’ relative to the median within their country. Remaining variables were coded, where appropriate, according to the needs of analysis and presentation of results. 

Response data from males and females were compared to examine differences in awareness parameters. Differences within each gender according to age, educational level and SES were also explored. Detailed results are included in Figure S1 and Tables S1–S6 as online supplementary data. 

Cross-tabs were produced to identify differences in responses according to gender. The Chi-squared statistic was estimated to examine the statistical relevance of differences, with significance being set at *p* < 0.05. No adjustments were made for pairwise comparisons. Finally, logistic regression models were developed to compare the most important awareness indicators according to gender, adjusting for age, SES, educational level and whether or not the participant had suffered a previous heart attack. All analyses were performed using the SPSS statistical software package version 22.0. 

## 3. Results

### 3.1. Participant Characteristics

Overall, 52.2% of respondents were female, 65.7% were aged older than 45 years, 38.3% had undertaken higher educational studies and 41.1% had a high relative SES. Participants’ demographic characteristics are presented in Table 1 both overall and according to gender. Female respondents were more likely than male respondents to belong to the older age group and be divorced/separated or widowed. They were less likely to report having received higher education (35.5% versus 41.2%) and having a high SES (36.6% versus 45.9%). Male respondents were more likely to have had a heart attack (6.7% versus 4.0%) and have been diagnosed with a cardiovascular disease different to CAD (12.0% versus 8.9%).

### 3.2. Awareness of Heart Disease

A minority of males (19.2%) and a significantly lower percentage of females (4.2%) considered heart disease to be a leading health issue, as shown in the results presented in Table 2. Sex differences were even greater in relation to perceptions of cardiovascular disease as a leading cause of death (49.9% men vs. 16.5% women).

Gender inequalities were also examined in relation to SES and educational level (Table 2) and age (Tables S1–S3 of supplementary material). Perception of heart disease as a leading health issue and as a leading cause of death increased with age within both males and females. Further, greater awareness of heart disease as a leading health issue was seen amongst those with a high SES compared with those with a low SES (22.7% vs 14.2% for males; 6.7% vs. 3.0% for females), as was greater awareness of heart disease as a leading cause of death (59.4% vs. 44.2% for males; 19.7% vs.15.2% for females). 

These inequalities were starker within men. Differences between the two educational groups only emerged within women in relation to heart disease as a leading health issue, whilst for men such differences only emerged in relation to heart disease as a leading cause of death. Complete results on these inequalities are included as supplementary material. 

### 3.3. Warning Signs of a Heart Attack and Calls to Emergency Services

Table 2 presents differences in awareness of the warning symptoms of a heart attack and participants’ initial response to the hypothetical experience of one. Chest pain/discomfort was the main warning sign reported, regardless of gender. Of the atypical warning signs, females were more likely to report nausea (12.6% vs. 5.3%) and palpitations (10.1% versus 8.0%). Only a small proportion of men and women were capable of spontaneously identifying the most common symptoms of a heart attack outside of pain (dyspnea, unusual fatigue, dizziness or generalised weakness) with no significant differences according to sex (16.3% males vs. 15.4% females). Slightly more than half of respondents would call emergency services in the event of a heart attack. Women were more likely to report this response than men (57.6% vs. 54.4%).

Knowledge of common warning signs decreased significantly with age. This knowledge was significantly higher within those with a high SES relative to those with a low SES and within those with a high educational level relative to those with low education. This was true for both males and females. In the event of a heart attack, the percentage of those who would call emergency services decreased slightly with age. Within women, differences based on educational level and SES were significant (education: 67.2% [high] vs 53.3% [low], SES: 64.7% [high] vs 51.9% [low]). Within men, no significant differences emerged according to SES whilst differences were much smaller in relation to educational level. Full results can be consulted in the supplementary material.

### 3.4. Perceived Risk Factors and Heart Disease Prevention Strategies

Table 3 presents participant perceptions of the main risk factors and preventive actions. Overall, stress was the most commonly reported risk factor (63.3%), with no significant gender differences. However, significant gender differences were found for most of the reported risk factors. Females were more likely than males to report obesity and less likely to report smoking, not exercising, and drinking alcohol.

Of the preventive actions, the most commonly cited, regardless of gender, was engaging in physical activity (67.0%). A number of significant gender differences were found, with females being more likely to report eating fruit and vegetables, having regular medical check-ups and engaging in hypertension control practices. In contrast, males were more likely to report maintaining a healthy weight, not smoking and better stress management. 

In general, a greater proportion of those belonging to younger age groups tended to accurately report the top five risk factors and the main preventive strategies. An exception to this was found for stress, with differences not being significant. Men and women with higher SES or with a higher educational level were systematically more able to identify main risk factors such as drinking alcohol, following an unhealthy diet, smoking and not exercising.

With regards to preventive actions, a greater proportion of men and women with a high educational level or high SES reported engaging in physical activity and not smoking. In contrast, those with a low socio-economic status were more likely to report having regular medical check-ups.

### 3.5. At-Risk Individuals and Medical History

Table 3 presents perceptions about the types of individuals who are most at risk of suffering from heart disease. A very high percentage of respondents, regardless of gender, agreed with the statement that “highly stressed executive professionals are more prone to heart attacks”. Females were less likely than males to agree with the statements that “men have more heart disease than women” and that “probability of heart disease in women increases after menopause”, and more likely to agree that “young women, under 50, do not have heart attacks”.

Table 3 also presents inequalities pertaining to medical history according to SES and educational level. A significantly higher proportion of those with low SES or with a low educational level had previously had a heart attack, with differences being particularly large amongst women. Differences in having had a screening test on CHD risk were not systematic. The proportion of both men and women to have taken screening tests increased with advancing age. Within men, differences were significant when stratifying according to SES but not when stratifying according to educational level. Within women, the opposite was found with a greater proportion of screening tests being taken by those with a low SES and low educational level than those with a high SES (36.9% vs. 32.1%) and high educational level (37.6% vs. 32.1%), respectively.

### 3.6. Key Awareness Indicators

**Figure 1:**
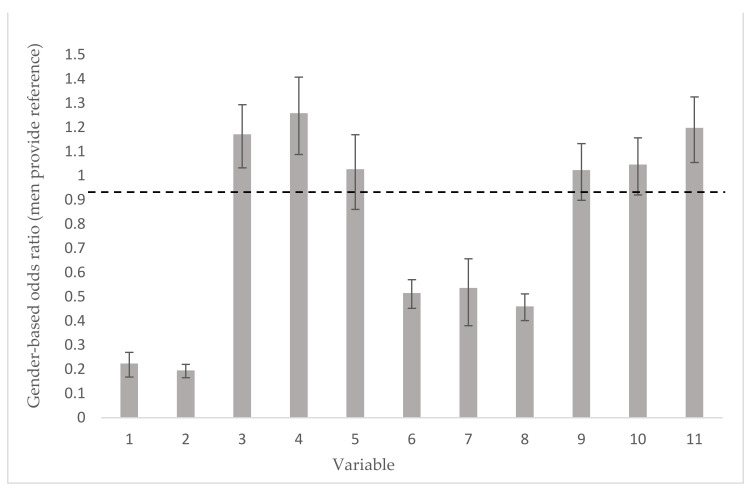
Odds Ratios and 95% CIs pertaining to selected indicators of awareness. Gender odds ratios and associated 95% CIs adjusted for age, SES, education and prior experience of a heart attack (males provide reference group), with the exception of variable 7 which is adjusted for age, SES and education. Dashed line is OR = 1, i.e., no gender difference. Weighted data. (1) Heart diseases is a main health issue for your gender; (2) Heart diseases is a leading cause of death for your gender; (3) Would call emergency services when suffering a heart attack; (4) Would call emergency services if somebody else suffered a heart attack; (5) Knows main warning signs of a heart attack; (6) Has taken a cardiovascular screening test ever; (7) Has suffered a heart attack; (8) Doctor has discussed risk factors; (9) Would like more information on heart disease; (10) I am informed about heart disease and the risk factors associated with it; (11) Knows what to do and how to do it, when it comes to preventing heart disease. *p* < 0.05 for variables 1–4, 6–8 and 11.

Table S6 of supplementary material shows the odds ratios predicting awareness of 11 key indicators in women relative to men, following adjustment for age, SES, educational level and whether one had suffered a previous heart attack. These odds ratios are presented in Figure 1. Results showed that women were approximately five times less likely to consider heart disease as the greatest health issue or leading cause of death amongst women (OR = 0.224, 95% CI: 0.178–0.280, OR = 0.196, 95% CI: 0.171–0.226). Further, women were significantly less likely to have discussed risk factors with their doctors (OR = 0.460, 95% CI: 0.408–0.518) and have ever taken a cardiovascular screening test. No gender differences were found with regards to knowledge of the main signs of a heart attack, although women were somewhat more likely to call emergency services than men should they suffer a heart attack. Finally, women in the present sample were significantly less likely to have had a heart attack (OR = 0.536, 95% CI: 0.415–0.691).

## 4. Discussion

The present study shows that levels of awareness in relation to CAD in this sample of the European population are far from adequate. Further, the study reveals that women have systematically lower awareness than men for many of the studied aspects. Whilst it is true that CVD tends to develop at a later age in women [16], it has similar epidemiological outcomes in men and women and, yet women gave it much less importance than men. This is made worse by the fact that significant social inequalities also emerged in key aspects of awareness of cardiovascular health. This was despite the fact that the studied population was familiar with heart disease, as evidenced by the high percentage reporting having personally suffered a CVD or having a family member or close friend who had. 

Awareness that heart disease is a major health issue was notably low and the majority of individuals also failed to recognize it as a leading cause of death. Similarly, a study carried out in five European countries almost two decades ago found that less than half of participants were able to correctly identify CHD as the leading cause of death [17]. It seems, therefore, that no major changes in CHD awareness amongst the European general public have occurred, despite the large expansion of medical, epidemiological and preventive knowledge about this disease over the last 20 years. In the present study, awareness of CHD as an important health issue or as the leading cause of death was much lower amongst women. Women attribute much less importance to heart disease than it warrants due to the epidemiological significance of related pathologies within women. This is especially true when we consider that CVD mortality amongst women in Europe is higher than their combined mortality from all cancers [1].

It is also of interest that when comparing present results with the results of reference studies from the USA, it can be seen that the percentage of present respondents aware that CHD is the leading cause of death is well below the percentage seen amongst American women [9]. As is the case in other studies, our results show that gender inequalities are further exacerbated by inequalities according to SES, education and age [18]. Moreover, in the present study, the outcome of combining inequalities in gender and SES was alarming. For instance, the percentage of low SES women who considered CHD to be a leading health issue (3.0% vs. 22.7%) or a leading cause of death (15.2% vs. 59.4%) was seven and four times lower, respectively, than that of high SES men.

For the population as a whole, CAD awareness is essential for the prevention of cardiovascular disease and associated mortality. The fact that women give greater relevance to other health problems with much less epidemiological significance suggests that gender specific effective strategies are needed [5,18].

Pain is the most cited symptom of a heart attack, being reported by just over half of those surveyed. This result is similar to that produced in other studies [18,19,20,21,22]. Although respondents were less likely to identify other typical heart attack symptoms, as was the case in other studied populations, they did identify the most important ones [17,22,23,24]. Exploring this further, women identified some of the atypical symptoms somewhat more frequently than men, although differences were small. The lack of differences in relation to identified symptoms is important as it places women at a disadvantage when it comes to recognizing and acting in the event of a heart attack since they tend to present with atypical symptoms [25,26].

In any case, spontaneous knowledge about the set of symptoms that characterize a heart attack is very low, being even lower in the present study than in studies of other European or American populations. These differences between studies may be due to the fact that the present study examined spontaneous responses, whereas other studies were based on predefined lists of symptoms [23]. Present results show that older people were less able to identify the symptoms of a heart attack. Results of previously published studies are not consistent in this regard [20,24,27]. Given that the vast majority of heart attacks occur at older ages, the consequences of this lack of knowledge could be important. In addition, correct symptom identification was poorer amongst people with a low educational level and low SES. This has also been found in other studies [14,19,24,27,28]. In the event of a heart attack, slightly more than half of participants would call emergency services. This percentage was somewhat higher within women and those belonging to younger age groups. Following adjustment for sociodemographic characteristics and previous heart attack history, the likelihood of calling emergency services was significantly higher amongst women. In addition, this percentage was significantly lower within men and women with a low educational level and SES [20,22,27].

Lack of knowledge of heart attack symptoms was associated with a failure to engage in the recommended action and call emergency services when suffering a heart attack [20]. Social inequalities both in the recognition of symptoms and in calling emergency services may significantly contribute to greater delays in receiving appropriate health care [29,30]. This could, therefore, contribute to the already known social inequalities in heart attack survival [31].

Both males and females reported stress to be the main risk factor for CHD, which is in itself relevant for cardiovascular health [32]. Lifestyle risk factors such as smoking, diet and not engaging in physical activity were more recognized than clinical factors. Signaled preventive activities reasonably correspond with prioritized risk factors and these results, in general, agree with those of previous studies and are in line with scientific evidence and with European recommendations [10,14,16,33].

Furthermore, differences emerged in relation to age. Both men and women were less likely to identify lifestyle risk factors with increasing age. Findings around the underestimation of this risk with age have already been described in women. Previous studies have also described how the percentage of women who identify clinical measures as main preventive measures, such as hypertension and diabetes control, increases with age [5,10].

SES and educational level are important factors when it comes to demonstrating inequality in the knowledge of risks and preventive measures [5,14,34]. Present results show that individuals with a high SES and educational level identified more risk factors and more preventive actions. In some cases, the magnitude of these inequalities was very significant. However, outcomes in this regard were not entirely unequivocal. For example, women with high education and SES identified physical activity in a very prominent way, whilst women with low education or SES gave more relevance to the consumption of fruit and vegetables, with inequalities disappearing in relation to this response. This could be due to differences in awareness, subjective perceptions of personal risk or existing barriers to the adoption of preventive actions [5,10,14,35].

More than 60% of men and women considered engaging in physical activity to be the main preventive activity, followed by consumption of fruits and vegetables in women, and smoking cessation in men. Clinical measures were indicated by less than a third of participants, with more women than men opting for such responses. The preventive actions indicated by the present population are in line with scientific evidence and with recommendations included in European guidelines [16,33]. However, stress control is not considered as a relevant preventive action, despite being identified by participants in the present study as the main risk factor for CHD. This may be due to the fact that individuals feel that stress is beyond their personal control as it is associated with adverse life conditions. In other words, the strong impact of social health determinants on stress means that social and community strategies are needed to control it [36,37].

The reasons behind women’s lower awareness is likely to be multi-factorial [38]. We have already pointed out the misconceptions about epidemiological relevance of cardiovascular disease among women, and that they give greater importance to other health problems. We have also noted the lack of gender-specific strategies to increase awareness. Additionally, results of the present study also show that doctors are less likely to discuss CHD risk with women than with men, possibly due to misperceptions that young women are not at risk [39]. This may be important in determining sex differences in awareness [40] as it deprives women of highly relevant information which could assist them in incorporating positive changes into their lifestyles. 

Furthermore, over 80% of participants, irrespective of gender, agreed that “men who are highly stressed executive professionals” are especially prone to heart attacks. Such a belief exists despite having no basis in any available scientific evidence [36], denoting a clear gender and class bias in relation to CHD risk. On the other hand, the fact that almost 70% of women knew that the risk of CHD increases with menopause, regardless of age, is a much more positive finding, although small inequalities did emerge according to educational level and SES.

The present study has some strengths and limitations. The study employed a representative sample of the European population. Nonetheless, the European Union is made up of a large number of countries which are highly heterogeneous in relation to key aspects, including cardiovascular health indicators, and the sampling approach used resulted in some countries being over-represented relative to others. Further, only a limited number of countries were considered although criteria used to select the countries from which the sample of participants was drawn were based on the aspects believed to be the most important to reflect. In addition, the approach taken enabled gender differences to be analyzed, unlike in most studies on awareness. This allowed us to examine outcomes in relation to the general population, and to women in particular. Finally, the survey entailed a validated questionnaire which was administered via telephone for feasibility reasons. A methodologically sound approach was taken, employing cutting-edge technology and managed by one of the most assured companies on the continent.

## 5. Conclusions

In conclusion, awareness of CAD is essential for individuals to be able to adopt preventive actions and healthy behaviors, identify main heart attack symptoms and reach emergency services on time. We have shown that the awareness of the European population needs improvement. Campaigns run in other countries have been identified to have produced good outcomes and present findings call for their further implementation [10,41]. More importantly, given the European advantages of having universal health care systems integrated within public health policies [42], a strategy should be developed based on the strong implementation of primary care which includes institutional policy committed to gender equality. This could be key to reducing social and gender inequalities in health [43].

## Figures and Tables

**Table 1:** Demographic characteristics of respondents to the CAD awareness survey, according to gender.

	Male		Female		Overall	
Characteristic	N	%	N	%	N	%
Gender	1252	47.8	1357	52.2	2609	100
Age						
25–44 years	425	34.0	469	34.6	895	34.3
45–64 years	514	41.0 *	513	37.8 *	1027	39.4
65 and over	313	25.0 *	374	27.6 *	687	26.3
Missing	0		0		0	
Health status						
Very good	261	20.8	280	20.6	541	20.7
Good	637	50.9	686	50.5	1323	50.7
Fair	272	21.8 *	325	23.9 *	597	22.9
Bad	65	5.2 *	52	3.8 *	117	4.5
Very bad	17	1.3	14	1.0	31	1.2
Missing	0		0		0	
Marital status						
Single	252	20.2	170	12.6	422	16.2
Married/living together	860	69.1 *	894	66.1 *	1754	67.5
Divorced/separated	70	5.6 **	132	9.8 **	203	7.8
Widow	63	5.1 **	157	11.6 **	220	8.5
Missing	0		0		0	
Education						
Compulsory or less	725	58.8 **	860	64.5 **	1584	61.7
Higher education	509	41.2 **	473	35.5 **	982	38.3
Missing	19	1.4	24	1.7	43	1.6
Relative socioeconomic status						
High	521	45.9 **	435	36.6 **	956	41.1
Low	614	54.1 **	752	63.4 **	1366	58.9
Missing	117	9.3	170	12.5	287	11.0
Health insurance						
Has health coverage	1219	97.4 *	1337	98.5 *	2556	98.0
Don’t know/No answer	12	1.0	10	0.7	22	0.8
Medical history						
Has suffered from a heart attack	83	6.7 **	54	4.0 **	137	5.3
Don’t know/No answer	1	0.1	1	0.1	2	0.1
Has been diagnosed with a cardiovascular disease (other than heart attack)	148	12.0 **	120	8.9 **	268	10.3
Don’t know/No answer	13	1.0	6	0.4	19	0.7
Close relative or friend has had a heart attack	731	58.7 *	843	62.5 *	1575	60.7
Don’t know/No answer	15	1.2	14	1.0	29	1.1
Close relative or friend has had another severe cardiovascular disease (different from a heart attack: myocardial infarction or angina pectoris)	502	40.9 *	589	44.2 *	1090	42.6
Don’t know/No answer	44	3.5	41	3.0	85	3.3
Have you ever taken a screening test to know about your risk of being affected by cardiovascular disease?	645	52.0 **	472	35.2 **	1134	43.2
Don’t know/No answer	13	1.0	14	1.0	27	1.0

Weighted data; * *p* < 0.05. ** *p* < 0.001.

**Table 2:** Awareness of selected leading health issues, warning signs of a heart attack and responses to signs of a heart attack, according to gender, education and SES.

					High Education				Low Education				High SES				Low SES			
	Male		Female		Male		Female		Male		Female		Male		Female		Male		Female	
Characteristic	N	%	N	%	N	%	N	%	N	%	N	%	N	%	N	%	N	%	N	%
Leading health issue (affecting your gender)																				
Cancer in general	250	22.8 *	235	19.9 *	119	26.4 ^x^^x^	76	18.2	131	20.3 ^x^^x^	159	21.0	116	22.3	71	16.4 ^††^	133	21.7	151	20.1 ^††^
Lung cancer	19	1.8 *	1	0.1 *	7	1.6	1	0.1	12	2.0	0	0.1	8	1.7	0	0.0	11	1.9	1	0.1
Breast cancer			266	22.5			92	22.2			174	22.9			74	17.2 ^††^			192	25.7 ^††^
Diabetes	30	2.8	28	2.4	12	2.8	12	2.9	18	2.8	16	2.2	14	2.7	13	2.9	16	2.8	15	2.1
Heart disease/Heart attack	210	19.2 **	50	4.2 **	88	19.7	25	5.9 ^††^	122	19.2	25	3.4 ^††^	118	22.7 ^x^^x^	28	6.7 ^††^	87	14.2 ^x^^x^	22	3.0 ^††^
Obesity	109	10.0 *	69	5.8 *	54	12.0 ^x^^x^	31	6.8 ^††^	55	8.8 ^x^^x^	38	4.7 ^††^	57	11.2 ^x^^x^	39	9.0 ^††^	52	8.5 ^x^^x^	26	3.4 ^††^
Don’t know/No answer	157	12.5	174	12.8	65	12.8	53	11.3	86	11.9	115	13.4	54	10.3	37	8.4	86	14.0	100	13.3
Leading cause of death (affecting your gender)																				
Accidental death	31	2.7 *	5	0.4 *	9	1.9 ^x^^x^	3	0.8 ^††^	22	3.3 ^x^^x^	2	0.2 ^††^	16	3.3	3	0.9 ^††^	15	2.8	2	0.3 ^††^
Cancer in general	260	22.9 *	573	49.1 *	101	21.4	202	48.7	159	23.7	371	49.2	100	19.1 ^x^^x^	218	50.1	146	23.8 ^x^^x^	355	47.4
Lung cancer	42	3.7 *	13	1.1 *	6	1.2 ^x^^x^	0	0.0 ^††^	36	5.2 ^x^^x^	13	1.8 ^††^	7	1.4 ^x^^x^	1	0.1 ^††^	29	4.7 ^x^^x^	11	1.4 ^††^
Breast cancer			199	17.0			72	17.1			127	16.7			68	15.7			129	17.1
Heart disease/Heart attack	565	49.9 **	192	16.5 **	259	55.5 ^x^^x^	75	18.1	306	46.1 ^x^^x^	117	15.6	301	59.4 ^x^^x^	83	19.7 ^††^	264	44.2 ^x^^x^	109	15.2 ^††^
Stroke	58	5.1	56	4.8	17	3.8 ^x^^x^	17	4.2	41	6.1 ^x^^x^	39	5.3	18	3.7 ^x^^x^	13	2.9^††^	40	6.9 ^x^^x^	43	6.0 ^††^
Don’t know/No answer	121	9.7	189	13.9	42	8.3	52	11.0	73	10.0	132	15.3	48	9.3	62	14.3				
What are the warning signs that you associate with having a heart attack?																				
Chest pain (discomfort and sharp pain)	759	60.6 **	779	57.4 **	334	65.6 ^x^^x^	305	64.4 ^††^	418	57.6 ^x^^x^	467	54.3 ^††^	340	65.3 ^x^^x^	288	66.3 ^††^	345	56.2 ^x^^x^	399	53.0 ^††^
Radiation of pain	532	42.5 **	659	48.6 **	221	43.5	246	52.0^†^	303	41.8	405	47.1 ^†^	237	45.4 ^x^^x^	241	55.4 ^††^	234	38.1 ^x^^x^	338	45.0 ^††^
Dyspnea (shortness of breath)	219	17.5 *	268	19.7 *	97	19.1	96	20.4	122	16.8	169	19.7	111	21.3 ^x^^x^	111	25.6 ^††^	89	14.5 ^x^^x^	129	17.2 ^††^
Nausea	66	5.3 **	171	12.6 **	36	7.0 ^x^^x^	68	14.4	29	4.0 ^x^^x^	102	11.9	36	7.0 ^x^^x^	42	9.7 ^†^	22	3.6 ^x^^x^	102	13.6^†^
Sweating	110	8.8 **	55	4.1 **	51	10.1	22	4.6	59	8.1	31	3.6	58	11.1 ^x^^x^	17	3.9	39	6.4 ^x^^x^	33	4.4
Unusual fatigue	60	4.8	64	4.7	18	3.6 ^x^	29	6.2 ^†^	42	5.8 ^x^	34	4.0 ^†^	20	3.9 ^x^	20	4.7	36	5.8 ^x^	38	5.1
Dizziness	180	14.4 *	160	11.8 *	95	18.6 ^x^^x^	59	12.4	85	11.8 ^x^^x^	99	11.5	77	14.7	56	12.8	88	14.4	91	12.1
Generalized weakness	83	6.7 *	62	4.6 *	38	7.4	22	4.7	45	6.3	40	4.7	33	6.3	22	5.0	45	7.4	39	5.2
Palpitations	100	8.0 *	138	10.1 *	45	8.9	48	10.1	52	7.2	84	9.8	35	6.8 ^x^	48	11.1	58	9.4 ^x^	79	10.5
Don’t know/No answer	305	24.4	140	10.3	109	21.4	35	7.5	188	25.9	104	12.1	105	20.1	22	5.1	174	28.4	97	12.9
Knows the main symptoms	205	16.3	208	15.4	97	19.1 ^x^^x^	96	20.4 ^††^	105	14.5 ^x^^x^	108	12.6 ^††^	102	19.6 ^x^^x^	89	20.5 ^††^	86	14.0 ^x^^x^	102	13.6 ^††^
If you thought you were experiencing a heart attack, what is the first thing you would do?																				
Take an aspirin	10	0.8	16	1.2	6	1.2	7	1.4	4	0.6	6	0.7	7	1.3 ^x^^x^	3	0.7	2	0.4 ^x^^x^	10	1.3
Go to a hospital	117	9.3	135	9.9	39	7.6 ^x^^x^	37	7.8 ^††^	78	10.8 ^x^^x^	96	11.2 ^††^	39	7.4 ^x^^x^	31	7.2 ^††^	72	11.7 ^x^^x^	94	12.5 ^††^
Call a doctor	187	15.0 *	227	16.7 *	68	13.4 ^x^^x^	51	10.8 ^††^	117	16.1 ^x^^x^	166	19.3 ^††^	77	14.8	59	13.5 ^††^	91	14.9	149	19.8 ^††^
Call emergency medical services (112)	681	54.4 *	781	57.6 *	298	58.6 ^x^^x^	317	67.1^††^	376	51.8 ^x^^x^	458	53.3 ^††^	286	54.8	281	64.7 ^††^	327	53.3	390	51.9 ^††^
Call your spouse or a family member	109	8.7 *	94	6.9 *	51	10.1 ^x^^x^	36	7.6	57	7.9 ^x^^x^	56	6.5	49	9.4	33	7.5	52	8.4	47	6.3
Other	119	9.5 *	71	5.2 *	43	8.5	21	4.5	68	9.4	50	5.8	60	11.6 ^x^^x^	24	5.5	47	7.7 ^x^^x^	38	5.0
Don’t know	28	2.2	33	2.5	3	0.5 ^x^^x^	4	0.8 ^††^	25	3.4 ^x^^x^	28	3.3 ^††^	4	0.7 ^x^^x^	4	1.0 ^††^	23	3.7 ^x^^x^	24	3.2 ^††^
Call emergency services versus all other options	681	54.4 *	781	57.6 *	299	58.7 ^x^^x^	318	67.2 ^††^	376	51.8 ^x^^x^	458	53.3 ^††^	286	54.8	281	64.7 ^††^	327	53.3	390	51.9 ^††^

Main symptoms: chest pain, radiation of pain, dyspnea, unusual fatigue, dizziness or generalized weakness. To be considered correct respondents had to provide three of the aforementioned, of which at least one had to be chest pain or radiation of pain. * indicates overall comparisons; ^x^ indicates comparisons between male subgroups; ^†^ indicates comparisons between female subgroups. *, ^x^, ^†^ *p* < 0.05. **, ^x^^x^, ^††^ *p* < 0.001. Note: For comparisons in which *n* < 5, analysis was re-run using Fisher’s exact test producing similar outcomes. Weighted data.

**Table 3:** Main risk factors, preventive actions and individuals most at risk of cardiovascular disease, according to gender, education and SES.

					High Education				Low Education				High SES				Low SES			
	Male		Female		Male		Female		Male		Female		Male		Female		Male		Female	
Characteristic	N	%	N	%	N	%	N	%	N	%	N	%	N	%	N	%	N	%	N	%
Main risk factors for suffering cardiovascular disease																				
High blood pressure	142	11.4 **	251	18.5 **	81	16.0 ^x^^x^	109	23.0 ^††^	60	8.3 ^x^^x^	141	16.4 ^††^	67	18.8	58	13.4 ^††^	75	17.8	65	8.7 ^††^
Cholesterol (low good cholesterol or high bad cholesterol)	74	5.9 **	142	10.4 **	46	9.1 ^x^^x^	64	13.5 ^††^	28	3.9 ^x^^x^	74	8.6 ^††^	37	11.0	32	7.4 ^††^	37	9.4	29	3.8 ^††^
Family history of heart disease or stroke	128	10.2 **	211	15.6 **	65	12.8 ^x^^x^	108	22.8 ^††^	62	8.6^x^^x^	97	11.3 ^††^	69	18.7 ^x^	50	11.6 ^†^	59	13.4 ^x^	64	8.5^†^
Smoking habit	794	63.4 *	812	59.8 *	322	63.2	300	63.4 ^†^	455	62.7	499	58.0 ^†^	369	60.4^x^	304	69.8^††^	351	48.9 ^x^^x^	429	57.0 ^††^
Drinking alcohol	598	47.8 **	376	27.7 **	231	45.4	134	28.3	354	48.8	237	27.5	258	37.0 ^x^	147	50.6^†^	262	32.0 ^x^	229	45.3 ^†^
Diabetes	46	3.6 **	99	7.3 **	29	5.8 ^x^^x^	41	8.7 ^†^	17	2.3 ^x^^x^	58	6.7 ^†^	22	6.3	20	4.5^†^	24	5.7	21	3.0^†^
Unhealthy diet habits	489	39.1	544	40.1	223	43.8 ^x^^x^	225	47.6 ^††^	259	35.7 ^x^^x^	311	36.2 ^††^	230	44.2 ^x^	181	41.5 ^†^	221	36.0 ^x^^x^	272	36.2 ^†^
Not exercising	537	42.9 *	542	40.0 *	277	54.5 ^x^^x^	236	49.9 ^††^	258	35.6^x^^x^	296	34.4 ^††^	254	48.7 ^x^	226	51.9 ^††^	200	32.5 ^x^^x^	262	34.9 ^††^
Obesity	394	31.4 **	501	36.9 **	182	35.8 ^x^^x^	187	39.6 ^†^	210	28.9 ^x^^x^	296	34.4 ^†^	207	39.8 ^x^	187	43.1 ^††^	153	24.9 ^x^^x^	162	21.6 ^††^
Stress	804	64.2	847	62.4	313	61.4^x^	313	66.2 ^†^	473	65.3 ^x^	529	61.5 ^†^	353	67.7	288	66.3	403	65.6	475	63.1
Other	95	7.6 **	226	16.7 **	35	6.9	89	18.9 ^†^	59	8.2	134	15.6^†^	35	10.2 ^x^	51	11.7	60	15.0 ^x^	105	13.9
Don’t know	143	11.4	72	5.3	40	7.8	14	3.0	103	14.2	52	6.0	30	5.8	7	1.7	103	16.8	47	6.2
Preventive actions																				
Eating more fruit and vegetables	544	43.4 *	626	46.3 *	211	41.5	178	37.6 ^††^	324	44.7	437	50.8^††^	212	40.6	201	46.1	273	44.4	365	48.6
Physical activity	846	67.6	891	65.9	362	71.2^x^	339	71.6 ^††^	481	66.3 ^x^	543	63.1 ^††^	389	74.7 ^x^^x^	309	71.0 ^††^	395	64.4 ^x^^x^	483	64.2 ^††^
Regular medical check-ups	351	28.1 *	409	30.3 *	126	24.8 ^x^	152	32.1	220	30.3^x^	249	29.0	135	25.9 ^x^	120	27.5 ^†^	191	31.1 ^x^	237	31.5 ^†^
Keep a healthy weight	472	37.7 *	478	35.3 *	215	42.2 ^x^^x^	187	39.5 ^††^	247	34.0 ^x^^x^	278	32.3 ^††^	212	40.7 ^x^	161	37.0	213	34.7 ^x^	255	33.9
Not smoking	636	50.8 **	638	47.2 **	269	52.8	245	51.8 ^††^	360	49.6	378	44.0 ^††^	276	53.0 ^x^	223	51.3 ^††^	289	47.0 ^x^	316	42.0 ^††^
Improve stress management	334	26.7 **	321	23.8 **	145	28.4 ^x^	121	25.5	180	24.8 ^x^	200	23.4	148	28.5	120	27.5 ^††^	161	26.3	157	20.9 ^††^
Hypertension control	275	22.0 **	374	27.7 **	99	19.4 ^x^	130	27.4	171	23.6 ^x^	237	27.6	106	20.3	107	24.7 ^†^	136	22.2	217	28.8 ^†^
Taking vitamins	18	1.4 *	30	2.2 *	6	1.2	7	1.4 ^†^	9	1.3	23	2.7^†^	1	0.2 ^x^^x^	8	1.8	14	2.3 ^x^^x^	22	2.9
Taking antioxidants	12	0.9	15	1.1	4	0.7	5	1.1	8	1.1	10	1.2	2	0.4 ^x^	5	1.1	9	1.4 ^x^	10	1.3
Hormone replacement therapy	4	0.3	3	0.2	0	0.0^x^	0	0.0 ^†^	4	0.5 ^x^	3	0.4 ^†^	1	0.1	1	0.1	2	0.4	1	0.1
Diabetes control	117	9.3 *	108	8.0*	44	8.6	28	6.0 ^†^	73	10.0	77	8.9 ^†^	39	7.5 ^x^	21	4.9 ^††^	61	9.9 ^x^	74	9.9^††^
Don’t know	13	1.1	10	0.7	4	0.8	1	0.2 ^†^	9	1.3	9	1.0 ^†^	2	0.3^x^	3	0.7	10	1.7 ^x^	3	0.4
Individuals who are most at risk of suffering from cardiovascular disease																				
Men have more heart diseases than women	960	80.2 **	892	69.9 **	417	82.0 ^x^	339	74.7 ^††^	542	78.8 ^x^	553	67.0 ^††^	443	85.1 ^x^^x^	312	71.7	461	75.0 ^x^^x^	517	68.8
Don’t know/No answer	55	4.4	81	6.0	29	5.7	24	5.0	26	3.6	54	6.3	24	4.7	14	3.3	21	3.4	44	5.8
Men that are highly stressed executive professionals are more prone to heart attacks	1059	85.7	1151	86.2	436	85.6	406	85.8	621	85.7	740	86.1	450	86.4	378	86.9	533	86.8	650	86.4
Don’t know/No answer	15	1.2	22	1.6	9	1.7	1	0.2	7	0.9	22	2.5	5	0.9	1	0.1	10	1.6	15	2.0
Young women, under 50, do not have heart attacks	310	25.7**	274	20.8 **	109	21.8 ^x^^x^	100	21.3	201	28.4 ^x^^x^	174	20.6	126	24.1 ^x^	78	17.9 ^†^	176	28.7 ^x^	165	21.9 ^†^
Don’t know/No answer	46	3.7	35	2.6	22	4.3	4	0.9	25	3.5	32	3.7	18	3.4	1	0.1	23	3.8	27	3.6
In women the probability of heart disease increases after menopause	667	65.2**	830	69.1 **	288	68.4 ^x^	320	75.1 ^††^	379	63.3 ^x^	510	65.8 ^††^	298	63.1	308	70.7	369	66.3	506	67.3
Don’t know/No answer	230	18.4	156	11.5	112	22.0	50	10.6	112	15.5	106	12.3	97	18.6	38	8.7	109	17.8	85	11.3
Only women who adopt behaviors and lifestyles of men will have heart disease	290	24.7 *	301	22.9 *	99	20.5 ^x^^x^	64	13.7 ^††^	191	27.9 ^x^^x^	237	27.9 ^††^	122	23.5 ^x^	72	16.6 ^††^	167	27.2 ^x^	199	26.5 ^††^
Don’t know/No answer	78	6.2	46	3.4	30	5.8	15	3.1	48	6.6	31	3.6	22	4.3	2	0.4	47	7.7	34	4.5
Only women which have brought up children will have heart disease	55	4.6	54	4.0	13	2.6 ^x^^x^	7	1.5 ^††^	42	6.1 ^x^^x^	45	5.2 ^††^	21	4.0	7	1.7 ^††^	28	4.6	43	5.7 ^††^
Don’t know/No answer	70	5.6	31	2.3	21	4.2	7	1.5	30	4.2	25	2.9	18	3.4	1	0.1	31	5.0	23	3.1

* indicates overall comparisons; ^x^ indicates comparisons between male subgroups; ^†^ indicates comparisons between female subgroups. *, ^x^, ^†^ *p* < 0.05. **, ^x^^x^, ^††^ *p* < 0.001. Weighted data.

## Data Availability

The data used for the study is available from the corresponding author upon request.

## Supplementary Materials

The following are available online at https://www.mdpi.com/article/10.3390/ijerph19031388/s1, Table S1: Demographic characteristics of respondents to the CHD awareness survey according to age, Table S2: Awareness of selected leading health issues, warning signs of a heart attack and responses to signs of a heart attack, according to age, Table S3: Main risk factors, preventive actions and individuals most at risk in relation to cardiovascular disease, according to age, Table S4: Demographic characteristics of respondents to the CHD awareness survey, according to socioeconomic status, Table S5: Demographic characteristics of respondents to the CHD awareness survey, according to education, Table S6: Prediction of selected indicators of awareness. Figure S1: Frequencies pertaining to selected indicators of awareness.
