# Supplementary material for: Gender and Social Inequalities in Awareness of Coronary Artery Disease in European Countries
Source: Int J Environ Res Public Health. 2022 Jan 26;19(3):1388. doi: 10.3390/ijerph19031388 (PMC8835179; doi:10.3390/ijerph19031388)
Supplement: Supplementary file 1 [file ijerph-19-01388-s001.zip › ijerph-1513116-supplementary.pdf]

SUPLEMENTARY MATERIAL

Table S1. Demographic characteristics of respondents to the CHD awareness survey according to **age**

	Overall			Males			Females		
	According to age								
Characteristic (%)	25-44	45-64	65+	25-44	45-64	65+	25-44	45-64	65+
**Gender**									
Female	52.6	50.2	54.7						
Male	47.4	49.8	45.3						
**Health status**									
Very good	30.2[a]	17.5[b]	13.1[c]	30.6[a]	17.7[b]	12.5[c]	29.8[a]	17.5[b]	13.5[c]
Good	53.7[a]	51.2[a]	46.3[b]	52.0	50.2	50.7	55.2[a]	52.1[a]	42.5[b]
Fair	13.2[a]	24.5[b]	33.1[c]	14.5[a]	23.5[b]	28.8[c]	12.1[a]	25.4[b]	36.8[c]
Bad	1.8[a]	5.8[b]	5.9[b]	1.7[a]	7.2[b]	6.7[b]	2.0[a]	4.5[b]	5.4[b]
Very bad	1.1	1.0	1.6	1.3	1.4	1.4	0.9[a,b]	0.6[b]	1.8[a]
**Marital status**									
Single	26.6[a]	13.3[b]	7.1[c]	36.3[a]	13.9[b]	9.1[c]	18.1[a]	12.7[b]	5.4[c]
Married/living together	68.8[a]	70.9[a]	60.7[b]	60.0[a]	74.0[b]	73.1[b]	76.7[a]	67.9[b]	50.4[c]
Divorced/separated	4.2[a]	11.8[b]	6.5[c]	3.3[a]	10.2[b]	1.3[c]	5.0[a]	13.4[b]	10.9[b]
Widow	0.4[a]	4.0[b]	25.7[c]	0.4[a]	1.9[b]	16.6[c]	0.3[a]	6.0[b]	33.3[c]
**Education**									
Compulsory or less	52.4[a]	69.8[b]	61.7[c]	53.8[a]	67.9[b]	50.6[a]	51.2[a]	71.7[b]	71.1[b]
Higher education	47.6[a]	30.2[b]	38.3[c]	46.2[a]	32.1[b]	49.4[a]	48.8[a]	28.3[b]	28.9[b]
**Relative socioeconomic status**									
High	50.5[a]	54.0[b]	78.0[c]	52.0[a]	49.6[a]	30.6[b]	47.3[a]	42.2[b]	15.0[c]
Low	49.5[a]	46.0[b]	22.0[c]	48.0[a]	50.4[a]	69.4[b]	52.7[a]	57.8[b]	85.0[c]
**Health insurance**									
Has health coverage	99.3[a]	99.9[b]	99.4[a]	98.8[a]	99.9[b]	99.4	99.9	99.9[b]	99.4[a]
**Medical history**									
Has suffered from a heart attack	1.1[a]	4.0[b]	12.5[c]	0.8[a]	5.8[b]	16.1[c]	1.4[a]	2.3[a]	9.4[b]
Has been diagnosed with a cardiovascular disease	4.9[a]	9.6[b]	18.6[c]	3.8[a]	13.8[b]	20.1[c]	5.9[a]	5.3[a]	17.4[b]
Close relative or friend has had a heart attack	58.3[a]	64.9[b]	57.4[a]	56.5[a]	61.8[b]	56.7[a]	60.0[a]	68.2[b]	57.9[a]
Close relative or friend has had another severe cardiovascular disease	37.1[a]	47.3[b]	43.0[c]	35.9[a]	43.4[b]	43.5[b]	38.1[a]	51.1[b]	42.5[a]
Have you ever taken a screening test to know about your risk of being affected by cardiovascular disease?	28.4[a]	50.1[b]	52.3[b]	33.8[a]	60.8[b]	62.3[b]	23.6[a]	39.5[b]	43.9[b]

Superscript letters indicate significant differences ($p < 0.05$). Different letters in adjacent cells indicate significant differences between groups.

Table S2. Awareness of selected leading health issues, warning signs of a heart attack and responses to signs of a heart attack, according to age.

	According to age								
	Overall			Males			Females		
Characteristic (%)	25-44	45-64	65+	25-44	45-64	65+	25-44	45-64	65+
**Leading health issue (affecting your gender)**									
Cancer in general	21.1	19.9^{a}	23.7^{b}	23.1	21.9	23.7	19.3^{a}	17.8^{a}	23.6^{b}
Lung cancer	0.4^{a}	1.2^{b}	1.1^{b}	0.9^{a}	2.3^{b}	2.2	0.1	0.1	0.1
Breast cancer	12.7^{a}	10.4^{b}	12.5				23.7	20.7	23.3
Diabetes	2.2	2.9	2.6	1.5^{a}	3.9^{b}	2.7	3.0	1.8	2.5
Heart disease/Heart attack	8.6^{a}	12.8^{b}	13.1^{b}	15.2^{a}	21.2^{b}	21.6^{b}	2.9^{a}	4.5	5.7^{b}
Obesity	7.8	8.7^{a}	6.5^{b}	10.2	9.8	9.9	5.7	7.4^{a}	3.6^{b}
**Leading cause of death (affecting your gender)**									
Accidental death	3.4^{a}	0.7^{b}	0.7^{b}	5.8^{a}	1.3^{b}	1.3^{b}	1.1^{a}	0.1^{b}	0.1^{b}
Alzheimer's	0.1^{a}	0.5^{b}	0.2	0.1	0.4	0.0	0.1	0.6	0.4
Lung cancer	1.7^{a}	2.5	3.0^{b}	2.9^{a}	3.1^{a}	5.8^{b}	0.6^{a}	1.9^{b}	0.4^{a}
Breast cancer	8.6	7.8^{a}	10.0^{b}				16.6	16.1	18.7
Cancer in general	39.9^{a}	36.0^{b}	32.1^{c}	26.2^{a}	23.1^{a}	18.5^{b}	52.6^{a}	49.5^{a}	44.0^{b}
Heart disease/Heart attack	26.9^{a}	36.4^{b}	34.9^{b}	41.9^{a}	53.4^{b}	54.3^{b}	12.8^{a}	18.6^{b}	18.1^{b}
Stroke	5.5^{a}	3.9^{b}	5.8^{a}	5.3^{a,b}	3.6^{b}	7.4^{a}	5.7	4.2	4.5
**What are the warning signs that you associate with having a heart attack?**									
Chest pain (discomfort and sharp)	57.1^{a}	61.5^{b}	57.5^{a}	56.7^{a}	64.4^{b}	59.9^{a,b}	57.4	58.6	55.6
Radiation of pain	44.1^{a}	49.6^{b}	41.8^{a}	37.6^{a}	48.4^{b}	39.4^{a}	49.9^{a}	50.9^{a}	43.7^{b}
Dyspnea (shortness of breath)	20.2^{a}	19.2^{a}	15.9^{b}	15.8^{a}	19.7^{b}	16.1	24.2^{a}	18.7^{b}	15.6^{b}
Nausea	6.3^{a}	12.3^{b}	7.9^{a}	4.1^{a}	7.5^{b}	3.5^{a}	8.3^{a}	17.1^{b}	11.6^{c}
Sweating	5.0^{a}	8.9^{b}	4.3^{a}	5.7^{a}	12.7^{b}	6.5^{a}	4.3^{a}	5.1^{a}	2.4^{b}
Unusual fatigue	5.3^{a}	3.6^{b}	5.8^{a}	4.9	3.8^{a}	6.4^{b}	5.6^{a}	3.4^{b}	5.4^{a}
Dizziness	17.8^{a}	11.3^{b}	9.4^{b}	20.8^{a}	11.9^{b}	9.6^{b}	15.0^{a}	10.7^{b}	9.4^{b}
Generalised weakness	8.1^{a}	4.4^{b}	4.1^{b}	9.7^{a}	5.1^{b}	5.2^{b}	6.7^{a}	3.7^{b}	3.2^{b}
Palpitations	11.3^{a}	9.5^{b}	5.7^{c}	10.7^{a}	7.6^{b}	5.0^{c}	11.8^{a}	11.4^{a}	6.3^{b}
**Knows the main symptoms**[1]	19.0^{a}	14.7^{b}	13.3^{b}	19.2^{a}	16.5^{a}	12.3^{b}	18.9^{a}	13.0^{b}	14.1^{b}
**If you thought you were experiencing a heart attack, what is the first thing you would do?**									
Take an aspirin	0.5^{a}	1.1	1.5^{b}	0.3^{a}	0.9	1.4^{b}	0.7	1.3	1.5
Go to a hospital	7.6^{a}	10.9^{b}	10.4^{b}	5.5^{a}	11.6^{b}	10.8^{b}	9.5	10.2	10.2
Call a doctor	14.8^{a}	15.2^{a}	18.4^{b}	14.8	14.8	15.6	14.8^{a}	15.5^{a}	20.8^{b}
Call emergency medical services (112)	58.3^{a}	55.7	53.6^{b}	58.6^{a}	51.3^{b}	54.0	58.2^{a}	60.1^{a}	53.3^{b}
Call your spouse or a family member	10.4^{a}	6.4^{b}	6.6^{b}	12.4^{a}	7.1^{b}	6.2^{b}	8.4^{a}	5.7^{b}	6.7
Other	6.4^{a}	8.3^{b}	6.8	7.1^{a}	11.5^{b}	9.6	5.8	5.2	4.5
Don't know	1.9	2.5	2.7	1.3^{a}	2.9^{b}	2.3	2.5	2.1	2.9
Call ambulance versus any other response	58.3^{a}	55.7	53.6^{b}	58.5^{a}	51.3^{b}	54.0	58.3^{a}	60.1^{a}	53.4^{b}

[1] **Main symptoms:** chest pain, radiation of pain, dyspnea, unusual fatigue, dizziness or generalized weakness. To be considered correct respondents had to provide three of the aforementioned, of which at least one had to be chest pain or radiation of pain.

Superscript letters indicate significant differences ($p < 0.05$). Different letters in adjacent cells indicate significant differences between groups.

Table S3. Main risk factors, preventive actions and individuals most at risk in relation to cardiovascular disease, according to age

	Age								
	Overall			Males			Females		
Response (%)	25-44	45-64	65+	25-44	45-64	65+	25-44	45-64	65+
**Main risk factors for suffering cardiovascular disease**									
High blood pressure	12.0^{a}	18.3^{b}	14.2^{a}	9.5	11.9^{a}	6.8^{b}	14.4^{a}	23.1^{b}	17.3^{a}
Cholesterol	7.8	8.8	8.2	6.5	4.4	5.8	8.4^{a}	13.0^{b}	9.4^{a}
Family history of heart disease or stroke	16.5^{a}	12.7^{b}	8.9^{c}	14.5^{a}	10.2^{b}	6.8^{c}	18.8^{a}	15.3^{b}	11.9^{c}
Smoking habit	59.0^{a}	67.7^{b}	55.6^{c}	54.8^{a}	60.2^{b}	47.8^{c}	58.8^{a}	66.4^{b}	52.2^{c}
Drinking alcohol	35.2^{a}	42.0^{b}	32.9^{a}	36.0^{a}	37.2^{a}	29.1^{b}	28.9^{a}	30.5^{a}	22.3^{b}
Diabetes	5.3	5.5	5.8	3.8	2.4^{a}	4.7^{b}	7.1	8.2	6.2
Unhealthy diet habits	49.0^{a}	37.6^{b}	30.4^{c}	45.3^{a}	35.9^{b}	26.6^{c}	52.2^{a}	37.3^{b}	28.9^{c}
Not exercising	48.6^{a}	40.0^{b}	34.0^{c}	41.4^{a}	39.4^{a}	27.2^{b}	48.6^{a}	36.7^{b}	33.6^{b}
Obesity	34.2^{a}	37.8^{b}	29.1^{c}	31.9^{a}	32.4^{a}	26.8^{b}	36.6^{a}	42.4^{b}	29.8^{c}
Stress	67.2^{a}	65.4^{a}	54.9^{b}	61.9^{a}	56.5^{b}	49.7^{c}	67.1^{a}	64.4^{a}	53.7^{b}
Other	14.2^{a}	11.4^{b}	11.3^{b}	13.3^{a}	9.7^{b}	6.8^{c}	9.0	11.0	12.3
**Preventive action**									
Eating more fruit and vegetables	46.2	44.1	44.1	45.4	41.9	43.1	47.0	46.3	44.9
Physical activity	72.7^{a}	68.2^{b}	56.2^{c}	74.6^{a}	66.8^{b}	59.2^{c}	70.9^{a}	69.6^{a}	53.7^{b}
Regular medical check-ups	34.7^{a}	26.9^{b}	25.3^{b}	28.8	27.3	28.2	40.1^{a}	26.4^{b}	22.9^{b}
Keep a healthy weight	36.0	37.2	35.7	37.2^{a}	41.6^{b}	31.7^{c}	34.9	32.8^{a}	39.0^{b}
Not smoking	49.7^{a}	50.8^{a}	44.9^{b}	52.1^{a}	53.7^{a}	44.3^{b}	47.4	47.8	45.4
Improve stress management	25.1	26.5	23.1	26.8	27.0	26.2	23.5	26.0^{a}	20.6^{b}
Hypertension control	18.3^{a}	24.5^{b}	34.1^{c}	16.8^{a}	21.3^{b}	30.0^{c}	19.5^{a}	27.7^{b}	37.6^{c}
Taking vitamins	1.8	2.1	1.5	2.7^{a}	0.9^{b}	0.6^{b}	0.9^{a}	3.2^{b}	2.5^{b}
Taking antioxidants	1.0	1.1	1.0	1.7^{a}	0.3^{b}	1.0^{a}	0.5^{a}	1.9^{b}	0.8^{a}
Hormone replacement therapy	0.3	0.1	0.4	0.7^{a}	0.1^{b}	0.1	0.0^{a}	0.1^{a}	0.6^{b}
Diabetes control	5.2^{a}	6.6^{b}	16.0^{c}	5.4^{a}	6.8^{a}	18.7^{b}	5.0^{a}	6.4^{a}	13.7^{b}
Don't know	0.8^{a}	0.5^{a}	1.6^{b}	0.5^{a}	0.8^{a}	2.3^{b}	1.0^{a}	0.2^{b}	1.1^{a}
**Individuals who are most at risk of suffering from cardiovascular disease**									
Men have more heart diseases than women	75.4	73.9	75.6	80.1	79.4	81.6	71.1	68.5	70.4
Men that are highly stressed executive professionals are more prone to heart attacks	86.7^{a}	84.2^{b}	87.7^{a}	87.6^{a}	83.2^{b}	87.3^{a}	85.9	85.2	88.0
Young women, under 50, do not have heart attacks	20.4^{a}	21.6^{a}	29.3^{b}	22.2^{a}	26.3^{b}	29.7^{b}	18.9^{a}	17.0^{a}	28.8^{b}
In women the probability of heart disease increases after menopause	65.9	67.0	69.6	63.9^{a}	63.1^{a}	70.6^{b}	67.5	70.5	68.8
Only women who adopt behaviors and lifestyles of men will have heart disease	17.8^{a}	23.4^{b}	32.5^{c}	21.3^{a}	22.3^{a}	33.6^{b}	14.7^{a}	24.5^{b}	31.5^{c}
Only women which have brought up children will have heart disease	3.5^{a}	3.8^{a}	6.2^{b}	5.1^{a}	3.1^{b}	6.4^{a}	2.0^{a}	4.4^{b}	6.1^{b}

Superscript letters indicate significant differences ($p < 0.05$). Different letters in adjacent cells indicate significant differences between groups.

Table S4. Demographic characteristics of respondents to the CHD awareness survey, according to **socioeconomic status**.

	Overall		Male		Female	
	Socioeconomic status					
Characteristic (%)	Low	High	Low	High	Low	High
**Gender**						
Male	44.7**	54.3**				
Female	55.3**	45.7**				
**Age**						
25-44 years	30.3**	42.5**	30.6^{xx}	38.9^{xx}	30.1^{xx}	46.7^{xx}
45-64 years	36.0**	43.9**	39.2^{xx}	45.3^{xx}	33.4^{xx}	42.2^{xx}
65 and over	33.7**	13.6**	30.3^{xx}	15.7^{xx}	36.5^{xx}	11.1^{xx}
**Health status**						
Very good	14.0**	30.9**	13.2^{xx}	29.8^{xx}	14.7^{xx}	32.3^{xx}
Good	50.8	51.3	51.5	51.4	50.3	51.1
Fair	26.9**	15.5**	24.4^{xx}	17.5^{xx}	29.0^{xx}	13.1^{xx}
Bad	6.4**	2.1**	8.6^{xx}	1.1^{xx}	4.7	3.3
Very bad	1.8**	0.2**	2.5^{xx}	0.2^{xx}	1.3^{xx}	0.2^{xx}
**Marital status**						
Single	20.8**	10.3**	27.4^{xx}	10.8^{xx}	15.5^{xx}	9.6^{xx}
Married/living together	55.3**	82.4**	56.8^{xx}	82.7^{xx}	54.0^{xx}	82.2^{xx}
Divorced/separated	11.4**	3.8**	7.7^{xx}	4.3^{xx}	14.5^{xx}	3.2^{xx}
Widow	12.5**	3.5**	8.1^{xx}	2.2^{xx}	16.1^{xx}	5.0^{xx}
**Highest educational level**						
Compulsory or less	74.1**	45.0**	71.3^{xx}	45.3^{xx}	76.4^{xx}	44.6^{xx}
Higher education	25.9**	55.0**	28.7^{xx}	54.7^{xx}	23.6^{xx}	55.4^{xx}
**Medical insurance**						
Has health coverage	99.4	99.8	99.1	99.7	99.7	99.9
**Medical history**						
Has suffered from a heart attack	6.8**	3.6**	7.5^{x}	5.5^{x}	6.1^{xx}	1.2^{xx}
Has been diagnosed with a cardiovascular disease (other than heart attack)	12.0**	7.6**	14.7^{xx}	7.9^{xx}	9.9^{x}	7.3^{x}
Close relative or friend has had a heart attack	59.3*	62.8*	59.1	61.5	59.5^{x}	64.5^{x}
Close relative or friend has had another severe cardiovascular disease	42.1	44.6	42.6	41.6	41.7^{x}	48.3^{x}
Have you ever taken a screening test to know about your risk of being affected by cardiovascular disease?	41.8**	45.1**	47.9^{xx}	55.9^{xx}	36.9^{x}	32.1^{x}

*,^{x} Denotes significant difference between adjacent columns at $p < 0.05$.

^{xx,}** Denotes significant difference between adjacent columns at $p < 0.001$

Table S5. Demographic characteristics of respondents to the CHD awareness survey, according to **education**.

	Overall		Male		Female	
	According to education					
Characteristic (%)	Compulsory or lower	Higher	Compulsory or lower	Higher	Compulsory or lower	Higher
**Gender**						
Male	45.6**	51.6**				
Female	54.5**	48.4**				
**Age**						
25-44 years	28.9**	42.4**	30.8[xx]	37.7[xx]	27.4[xx]	47.4[xx]
45-64 years	44.7**	31.2**	47.6[xx]	32.2[xx]	42.2[xx]	30.2[xx]
65 and over	26.4	26.4	21.6[xx]	30.2[xx]	30.4[xx]	22.5[xx]
**Health status**						
Very good	17.3**	26.3**	17.0[xx]	26.6[xx]	17.6[xx]	26.1[xx]
Good	48.2**	54.9**	51.0	51.7	46.0[xx]	58.3[xx]
Fair	26.9**	15.9**	23.3[xx]	18.2[xx]	29.9[xx]	13.5[xx]
Bad	5.9**	2.4**	6.8[xx]	3.1[xx]	5.2[xx]	1.7[xx]
Very bad	1.7**	0.4**	2.0[xx]	0.4[xx]	1.4[xx]	0.5[xx]
**Marital status**						
Single	14.1**	19.5**	19.3	21.2	9.7[xx]	17.8[xx]
Married/living together	67.3	67.6	69.5	68.6	65.6	66.6
Divorced/separated	9.6**	5.3**	6.9[x]	4.0[x]	11.8[xx]	6.6[xx]
Widow	9.1**	7.6**	4.4[x]	6.2[x]	13.0[xx]	9.0[xx]
**Health insurance**						
Has health coverage	99.5	99.6	99.2	99.6	99.8	99.7
Has suffered from a heart attack	6.4**	3.6**	7.2	6.1	5.7[xx]	1.0[xx]
Has been diagnosed with a cardiovascular disease	10.8*	8.7*	12.8	11.2	9.1[x]	6.1[x]
Close relative or friend has had a heart attack	61.9	60.0	59.8	57.8	63.7	62.3
Close relative or friend has had another severe cardiovascular disease	40.6**	46.2**	40.3	42.3	41.0[xx]	50.2[xx]
Have you ever taken a screening test to know about your risk of being affected by cardiovascular disease?	44.8*	41.8*	53.5	50.8	37.6[x]	32.1[x]

*,[x] Denotes significant difference between adjacent columns at $p < 0.05$.

[xx], ** Denotes significant difference between adjacent columns at $p < 0.001$

Table S6. Prediction of selected indicators of awareness

Characteristic (men provide the reference category)	OR	95% CI	p-value
Heart diseases is a main health issue for your gender*	0.224	0.178 - 0.280	.000
Heart diseases is a leading cause of death for your gender*	0.196	0.171 - 0.226	.000
Would call emergency services when suffering a heart attack*	1.171	1.048 - 1.309	.005
Would call emergency services if somebody else suffered a heart attack*	1.258	1.108 - 1.428	0.000
Knows main warning signs of a heart attack*	1.027	0.884 - 1.193	.726
Has taken a cardiovascular screening test ever*	0.515	0.459 - 0.578	.000
Has suffered a heart attack**	0.536	0.415 - 0.691	.000
Doctor has discussed risk factors*	0.460	0.408 - 0.518	.000
Would like more information on heart disease*	1.023	0.913 - 1.147	.691
I am informed about heart disease and the risk factors associated with it*	1.046	0.935 - 1.171	.431
Knows what to do and how to do it, when it comes to preventing heart disease*	1.198	1.070 - 1.341	.002

*Adjusted for age, SES, educational level and history of prior heart attack

** Adjusted for age, SES and educational level

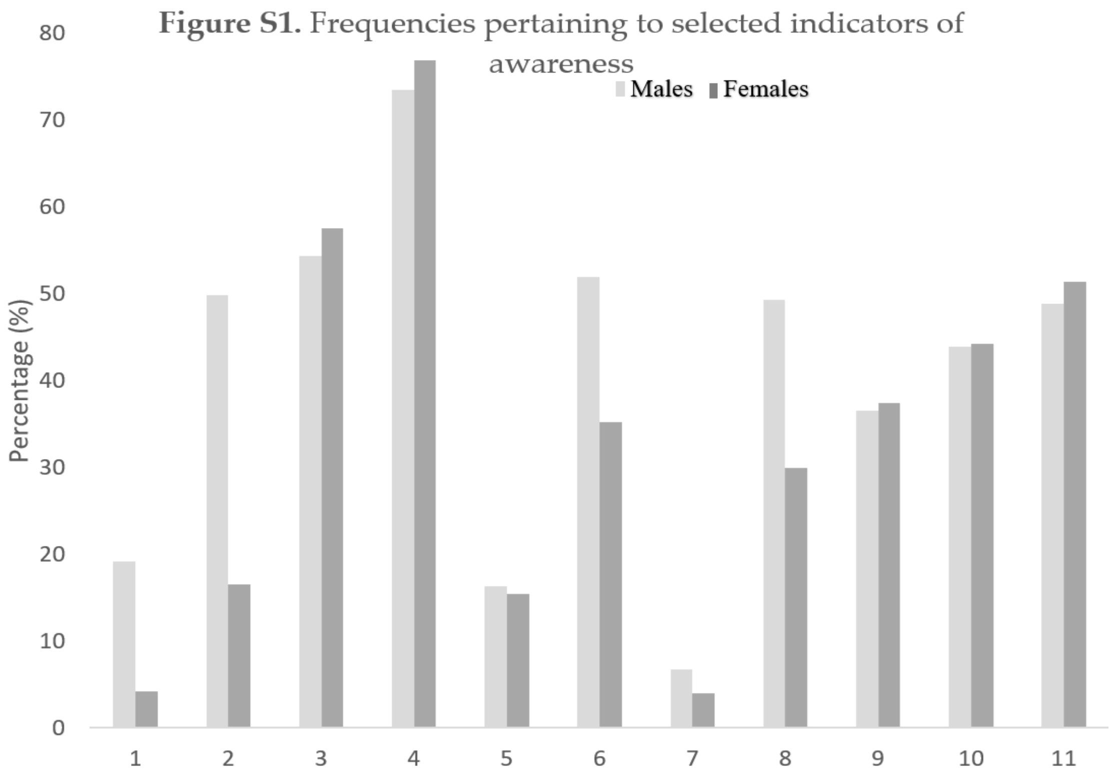

Figure S1. Bars represent weighted frequency of males and females reporting variables 1-11. 1) Heart diseases is a main health issue for your gender; 2) Heart diseases is a leading cause of death for your gender; 3) Would call emergency services when suffering a heart attack; 4) Would call emergency services if somebody else suffered a heart attack; 5) Knows main warning signs of a heart attack; 6) Has taken a cardiovascular screening test ever; 7) Has suffered a heart attack; 8) Doctor has discussed risk factors; 9) Would like more information on heart disease; 10) I am informed about heart disease and the risk factors associated with it; 11) Knows what to do and how to do it, when it comes to preventing heart disease.
$p < 0.05$ for variables 1-4, 6-8 and 11.
